# NeurphologyJ: An automatic neuronal morphology quantification method and its application in pharmacological discovery

**DOI:** 10.1186/1471-2105-12-230

**Published:** 2011-06-08

**Authors:** Shinn-Ying Ho, Chih-Yuan Chao, Hui-Ling Huang, Tzai-Wen Chiu, Phasit Charoenkwan, Eric Hwang

**Affiliations:** 1 Institute of Bioinformatics and Systems Biology, National Chiao Tung University, Hsinchu, Taiwan; 2 Department of Biological Science and Technology, National Chiao Tung University, Hsinchu, Taiwan; 3 Institute of Molecular Medicine and Bioengineering, National Chiao Tung University, Hsinchu, Taiwan

## Abstract

**Background:**

Automatic quantification of neuronal morphology from images of fluorescence microscopy plays an increasingly important role in high-content screenings. However, there exist very few freeware tools and methods which provide automatic neuronal morphology quantification for pharmacological discovery.

**Results:**

This study proposes an effective quantification method, called NeurphologyJ, capable of automatically quantifying neuronal morphologies such as soma number and size, neurite length, and neurite branching complexity (which is highly related to the numbers of attachment points and ending points). NeurphologyJ is implemented as a plugin to ImageJ, an open-source Java-based image processing and analysis platform. The high performance of NeurphologyJ arises mainly from an elegant image enhancement method. Consequently, some morphology operations of image processing can be efficiently applied. We evaluated NeurphologyJ by comparing it with both the computer-aided manual tracing method NeuronJ and an existing ImageJ-based plugin method NeuriteTracer. Our results reveal that NeurphologyJ is comparable to NeuronJ, that the coefficient correlation between the estimated neurite lengths is as high as 0.992. NeurphologyJ can accurately measure neurite length, soma number, neurite attachment points, and neurite ending points from a single image. Furthermore, the quantification result of nocodazole perturbation is consistent with its known inhibitory effect on neurite outgrowth. We were also able to calculate the IC50 of nocodazole using NeurphologyJ. This reveals that NeurphologyJ is effective enough to be utilized in applications of pharmacological discoveries.

**Conclusions:**

This study proposes an automatic and fast neuronal quantification method NeurphologyJ. The ImageJ plugin with supports of batch processing is easily customized for dealing with high-content screening applications. The source codes of NeurphologyJ (interactive and high-throughput versions) and the images used for testing are freely available (see Availability).

## Background

Recent advancements in automated fluorescence microscopy have made high-content screening an essential technique for discovering novel molecular pathways in diseases [1] or potential new therapeutic treatments [2,3]. However, high-content screenings on biological or pharmacological molecules that can induce neuronal differentiation, promote neuronal regeneration, or delay neurodegeneration are very limited. The main restricting factor is the lack of adequate tools for rapidly analyzing and quantifying the massive amount of neuronal images.

A neuron typically consists of two morphological structures, the round neuronal cell body (called soma) and the elongated neuronal protrusions (called neurites). To determine the efficacy of a particular pharmacological perturbation on neuronal regeneration using high-content screening techniques, automatic quantification of several morphological features is necessary. These features include soma number, soma size, neurite length, and neurite branching complexity. Although some of the small-scale screenings were conducted by manual quantification of neuronal morphology [4,5], these manual methods are extremely time-consuming and becoming impractical for large datasets. While commercially available software capable of automatic quantification of neurite outgrowth have been used in recent high-content screening studies [6-8], such tools are only available to large research facilities and are usually not openly available for user customization. These commercial software packages available for 2D or 3D neurite quantification include Amira (Visage Imaging), HCA-Vision (CSIRO Biotech Imaging), Imaris (Bitplane), and Neurolucida (MBF Bioscience).

Due to the limited budget of individual laboratories and various cell models and experimental designs amongst them, the open source codes of freeware tools are immensely useful for researchers. There are many freeware tools capable of quantifying neurite morphology, such as NeuronIQ [9], NeuronMetrics [10], NeuronJ [11], NeuronStudio [12], NeuriteIQ [13], NeuriteTracer [14], and NeuronCyto [15] for 2D applications; FARSIGHT [16], Neuromantic [17], Neuron_Morpho [18], and V3D [19] for 3D applications. For a comprehensive survey of recent developments in the field of neuron tracing, we recommend a recent review written by Erik Meijering [20]. Amongst the freeware tools, only four of them (NeuriteIQ, NeuriteTracer, NeuronCyto, and NeuronMetrics) possess high level automation needed for quantifying large volume of 2D images from a typical high-content screen. A comparison between NeurphologyJ and these four freeware toolkits is shown in Table 1.

**Table 1:** Free, open-source neurite quantification software packages for quantifying large volume of 2D fluorescence images

Name	Operation Mode	Morphology Measurements	Platform
NeuronMetrics [10]	Semi-automatic	Neurite length	ImageJ
		Soma number	
		Neurite complexity	

NeuriteIQ [13]	Automatic	Neurite length	Matlab
		Soma number and size	

NeuriteTracer [14]	Automatic	Neurite length	ImageJ
		Soma number	

NeuronCyto [15]	Automatic	Neurite length	Matlab
		Soma number and size	
		Neurite complexity	

NeurphologyJ	Automatic	Neurite length	ImageJ
		Soma number and size	
		Neurite attachment points	
		Neurite ending points	

ImageJ is an open-source Java-based image-processing and analysis platform [21]. It has quickly become one of the most popular image processing platforms due to its free availability, open-source nature, and large user community (and hence a variety of free plugins). For this reason, we designed our neuronal morphology quantification method based on ImageJ and compared it using two ImageJ-based toolkits (NeuronJ and NeuriteTracer).

NeuronJ plugin to ImageJ is the most popular computer-aided manual neurite tracing program and has been used as a reference tool for testing 2D neuron tracing algorithms [11]. Given each pair of starting point and ending point manually, the algorithm NeuronJ can extract the central line of neurites by finding an optimal path from the starting point to the ending point. The optimal path is found by calculating the globally minimal cumulative cost using a predefined cost function. Due to the nature of its design, NeuronJ is very accurate but extremely time-consuming. NeuriteTracer is another ImageJ plugin for automated neurite quantification capable of accurately processing large volume of 2D images [14]. Given user-defined thresholds, NeuriteTracer can estimate the neurite length which correlates strongly with that obtained manually using NeuronJ. It is important to note that a pair of images corresponding to nuclei (Hoechst 33342) and neurites (beta-III-tubulin) is required as the input of NeuriteTracer.

Most automatic quantification algorithms for measuring neurite outgrowth focus on estimating neurite length, which can be classified into two classes. Algorithms in the first class trace a series of points along the centreline of the segmented neurite from a detected seed point by estimating the local direction of each point [13,22-24]. The advantage of these algorithms is the accurate estimation of neurite length without further using linking algorithms. The disadvantage of these algorithms is the limited applications that only high-quality images with simple line structures are available. Algorithms in the other class detect pixels in line segments using local geometric properties of the lines such as ridges and ravines [14]. Generally, the Gaussian smoothing kernel is effectively utilized to extract line pixels by using the first and second derivatives of the line pixels. The advantages of algorithms in this class are both high processing speed and ability of dealing with uneven intensity images. Because the lines with low intensity contrast may be smoothened out by the Gaussian kernel, an additional linking procedure is needed for accurately estimating neurite length.

Here we describe an effective neuronal quantification method, called NeurphologyJ, capable of automatically quantifying neuronal morphology from large volumes of 2D fluorescent images that are generated in a typical drug screen. The automated tracing method NeuriteTracer and the computer-aided manual tracing method NeuronJ were used to evaluate the performance of NeurphologyJ. Our results reveal that NeurphologyJ performs well compared with NeuronJ and NeuriteTracer, and it can efficiently quantify the effect of nocodazole on inducing neurite retraction.

## Methods

### Neuron image acquisition

To evaluate whether NeurphologyJ can detect neuronal morphological changes upon pharmacological perturbation, we design an experiment to measure the effect of nocodazole on neurite length. Nocodazole is a known microtubule-destabilizing drug that has been shown to induce rapid neurite retraction when applied to neurons [25,26]. P19 neurons were incubated with increasing dosage of nocodazole for 24 hrs before being fixed and immunofluorescence stained. A total of 216 images (with a total size over 500 Mb) were analyzed using NeurphologyJ. The image acquisition procedure is described below.

#### a) Cell culture and drug treatment

Embryonic carcinoma P19 cells were maintained at 37°C in 5% CO2 in minimum essential medium supplemented with 2 mM glutamine, 1 mM sodium pyruvate, and 10% (v/v) fetal bovine serum. The drug experiment was performed on 96-well plates. Each well on the plate was pre-spotted with 800 ng of proneural gene (MASH1) expressing plasmid and 0.4 μL of Lipofectamine 2000 in a total of 50 μL serum-free minimum essential medium. After 20 minutes, 16000 P19 cells in differentiation medium (minimum essential medium supplemented with 2 mM glutamine, 1 mM pyruvate, 5% fetal bovine serum) were added to each well and maintain in a 37°C, 5% CO2 incubator. 72 hours post-transfection, P19 cell cultures were treated with DMSO (control) and various concentration of nocodazole (10, 50, 100, 200, and 1000 nM). After 24 hours of incubation, drug-treated cells were fixed with 3.6% formaldehyde in PBS. Fetal bovine serum was purchased from Biological Industries. Lipofectamine 2000, minimum essential medium, sodium bicarbonate, and trypsin-EDTA were purchased from Invitrogen. DMSO, nocodazole, and sodium pyruvate were purchased from Sigma-Aldrich.

#### b) Indirect immunofluorescence staining and image acquisition

Cells were fixed with 3.6% formaldehyde in PBS (prewarmed to 37°C) for 10 min at 37°C and permeabilized with 0.25% triton X-100 for 5 min at room temperature. Cells were blocked for 1 hr at room temperature with 10% BSA (bovine serum albumin), and incubated for 1 hr at 37°C with antibody against beta-III-tubulin (TUJ1) 1:4000 diluted in wash buffer (0.5% BSA, 0.05% tween-20 diluted in PBS). After being washed three times with wash buffer, cells were incubated with DyLight 488-labeled secondary antibodies (1:1000), and DNA-binding dye DAPI (5 μg/mL) for 1 hr at 37°C in the dark. Each well with cells were washed three times with wash buffer and stored in PBS. Formaldehyde and triton X-100 were purchased from J. T. Baker. BSA was purchased from Invitrogen. Mouse monoclonal antibody against beta-III-tubulin (TUJ1; MMS-435P) was purchased from Covence. DyLight 488-labeled goat-anti-mouse secondary antibody was purchased from Jackson ImmunoResearch. DAPI was purchased from Invitrogen. Fluorescence images were acquired with an Olympus IX-71 inverted microscope equipped with a CoolLED fluorescent light source (400 nm and 490 nm wavelength modules) and a Hamamatsu ORCA-R2 camera (6.45 μm × 6.45 μm pixel dimensions). Chroma BFP-A-Basic and Olympus U-MWIBA3 filter sets were used to image DAPI and DyLight488 fluorophores, respectively. Olympus Plan Apochromat objective lenses (10x 0.4 N.A. or 60x 1.35 N.A.) were used to collect images. A total of 216 images were taken and used for this experiment. The entire collection is over 500 Mb in size and can be downloaded from our FTP server upon request.

### Proposed method NeurphologyJ

The design aims of NeurphologyJ are as following.

1) Minimizing human intervention. It is essential to minimize the human intervention and the number of control parameters without degrading performance during batch processing. A translation of Occam's razor principle suggests that ending up with a large number of user-settable parameters is indicative of poor algorithm design [20]. An elegant image enhancement method is proposed to facilitate the determination of threshold values of segmentation.

2) Convenience of use. NeuriteTracer [14] is effective and accurate, but a pair of nuclear and neurite marker images is needed. It is more convenient if a single image of fluorescence microscopy is sufficient to measure neurite outgrowth. Only one channel per image is needed for applying NeurphologyJ.

3) Maximizing the speed. Considering the vast amount of images generated from the high-content screening, a high analyzing speed is crucial to handle such task. NeurphologyJ makes the best use of both global morphology operations of image processing and local geometric properties of lines to speed up the quantification.

4) Achieving high accuracy. There are tradeoffs between the processing speed and the accuracy. For applications in pharmacological discoveries, the ratio of neurite lengths of the treated and non-treated neurons (rather than the absolute neurite length) is the major concern. As a result, NeurphologyJ aims to achieve high coefficient correlation with manual tracing by detecting line pixels of neurites without further using linking algorithms.

5) Robustness. Image segmentation plays an important role in quantifying neuronal morphology. The techniques of local exploration and global processing are combined to deal with the staining or the illumination variation of the high-content screenings. Some settings of threshold values can be automatically derived from the histogram of enhanced neuronal images.

6) Taking advantage of the free software ImageJ. NeurphologyJ makes the best use of ImageJ commands and uses a compact set of Java modules. Being designed as a plugin of ImageJ has the benefit of easy customization for dealing with specific applications or for future expansions. Two versions of NeurphologyJ are provided, interactive and high-throughput. The interactive version is useful for optimizing the parameters for the high-throughput version.

**Figure 1:**
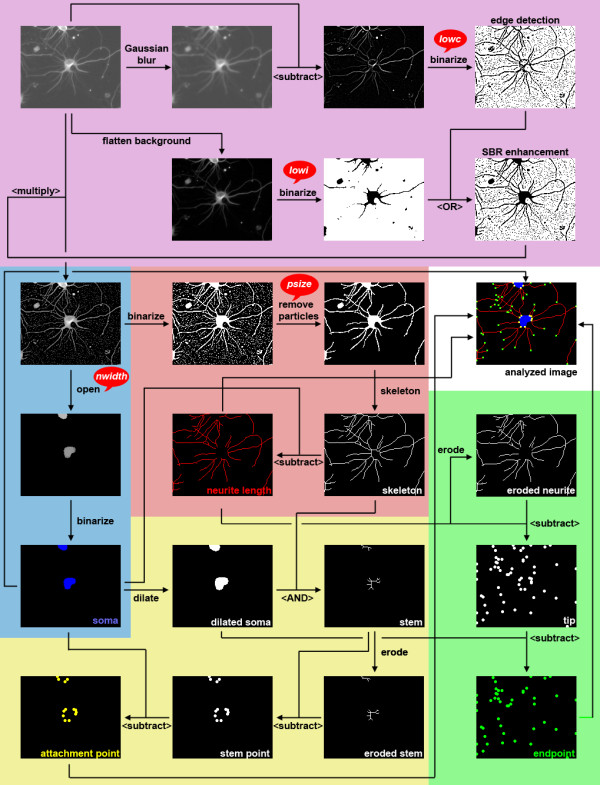
**The schematic flowchart of NeurphologyJ**. NeurphologyJ consists of five parts, each part is highlighted by a specific color. Arithmetic and logical operations are shown in <brackets> with logical operations capitalized.

The algorithm of NeurphologyJ consists of five parts, one image enhancement part and four morphological quantification parts. The schematic flowchart of NeurphologyJ is shown in Figure 1. The major commands used in each part and detailed descriptions are shown below.

### Image enhancement

**Figure 2:**
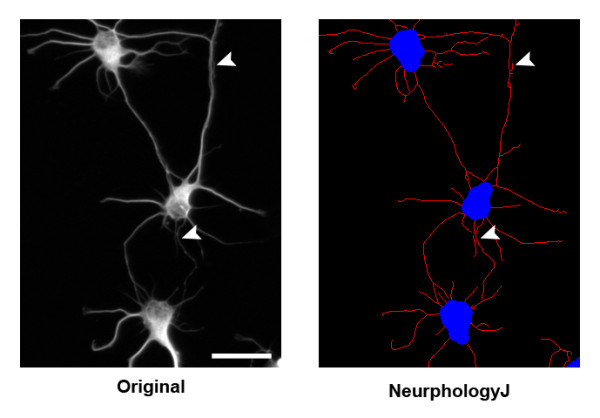
**Image enhancement process of NeurphologyJ does not remove thin and dim neurites**. Shown here is an example image of mouse hippocampal neurons analyzed by NeurphologyJ. Notice that both thick neurites and thin/dim neurites (arrowheads) are preserved after the image enhancement process. The scale bar represents 50 μm.

The image enhancement part is crucial for subsequent morphological quantifications. The proposed enhancement method aims to increase the signal-to-background ratio that can facilitate automatic determination of threshold values from the histograms of enhanced images. Furthermore, we examined and confirmed that thin and dim neurites are mostly preserved after this enhancement process using mouse hippocampal neuron images (Figure 2). The three functions used to achieve image enhancement are edge detection, uneven background correction, and intensity-based pixel selection. The detailed description is shown below.

**Figure 3:**
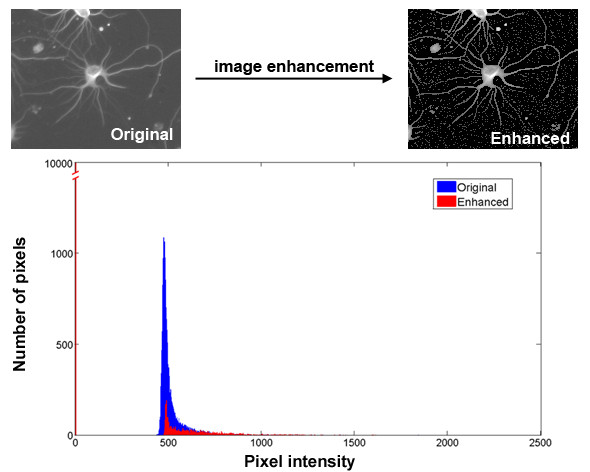
**The histograms of the original and the enhanced images**. After the enhancement, the background pixels of the original image were identified and their gray levels were set to zero (the red vertical line next to the Y-axis). The histogram of the original image is shown in blue and the enhanced image is shown in red. Notice that histogram stretching has been performed on both the original and the enhanced images for easy visualization.

1) To detect edges based on local intensity variation, the original image is subtracted by the image which has been smoothened by a Gaussian smoothing kernel. The resulting image is then binarized using a given threshold (*lowc*) to select pixels with low local contrast.

2) To correct the uneven background, the "Subtract Background" command using a rolling ball with a radius of *N *pixels (*N *is a constant 50 in this study) is applied to the original image. The flattened background image is binarized using a given threshold (*lowi*) to select low intensity pixels.

3) The gray levels of pixels selected by the first two steps (i.e., background pixels) are set to zero. These operations produce an image with increased signal-to-background ratio. Therefore, subsequent operations on foreground pixels can be easily done without background interference. Figure 3 shows the typical histograms of the original and the enhanced images.

This enhancement algorithm of NeurphologyJ aims to generate an enhanced image *I-new *from the original image *I*. All the features of neuronal morphology are extracted from the image *I-new*. Some typical images produced in the following steps are shown in Figure 1.

Step 1) Detecting low contrast pixels to generate an image *I-low_contrast*

1.1) *I-blur *= run("Gaussian Blur") on image *I*.

1.2) *I-sub_blurred *= imageCalculator("Subtract create", "*I*", "*I-blur*").

1.3) *I-low_contrast *= Binarize *I-sub_blurred *by setThreshold(0, *lowc*).

Step 2) Detecting low intensity pixels to generate an image *I-low_intensity*

2.1) *I-flatten *= run("Subtract Background") on image *I*.

2.2) *I-low_intensity *= Binarize *I-flatten *by setThreshold(0, *lowi*).

Step 3) Create a new image *I-new *by removing low contrast and low intensity pixels

3.1) *I-zero_intensity *= imageCalculator("OR create", " *I-low_contrast*", " *I-low_intensity*").

3.2) *I-new *= imageCalculator("Multiply create", " *I*", "*I-zero_intensity*").

The threshold values of *lowc *and *lowi *are manually determined by using the interactive version of NeurphologyJ. User-determined *lowc *and *lowi *values are reused in the high-throughput version for batch analysis.

### Soma extraction and quantification

From the enhanced image *I-new*, an Open operation (Erosion followed by Dilation operations) is used to isolate somata. The Open operation needs a parameter of the radius which equals the width of the thickest neurite (called the parameter *nwidth*). The value of *nwidth *is user-determined. This Open operation has an additional benefit of removing small contaminating objects such as cell debris. This "opened" image is then binarized for soma number and soma size quantification using the build-in command "Analyze Particles" of ImageJ.

Step 1) *I-open *= Using an Open operation on *I-new *to isolate neuronal cell bodies.

Step 2) *I-soma *= Binarize *I-open *by setThreshold(*Th1*, Gmax).

Step 3) Quantify soma pixels using "Analyze Particles".

The constant Gmax is the largest value of gray levels which is predefined, e.g., 255 for 8-bit images and 4095 for 12-bit images. Because the gray levels of background pixels have all been set to zero, the threshold value of *Th1 *is always set to 1.

### Neurite length extraction and quantification

The enhanced image is first binarized automatically and all cell debris and small particles are removed by a user-defined size (called the parameter *psize*). The resulting "cleaned" image is skeletonized to thin all objects into one-pixel-width skeletons. Somata are subtracted from the "skeleton" image to obtain the image presenting neurite length. Neurite length is quantified by counting all the pixels in the "neurite length" image using the "Analyze Particles" command.

Step 1) *I-neuritesoma1 *= Binarize *I-new *by setThreshold(*Th2*, Gmax).

Step 2) *I-neuritesoma2 *= run("Particle Remover") from *I-neuritesoma1*.

Step 3) *I-neuritesoma *= run("Skeletonize") on *I-neuritesoma2*.

Step 4) *I-neurite_length *= imageCalculator("Subtract create"," *I-neuritesoma*"," *I-soma*").

Step 5) Quantify neurite length using "Analyze Particles".

The threshold value of *Th2 *is similarly set to 1 (like *Th1*).

### Attachment point extraction

We defined the neurite attachment point as the location where neurite connect to the soma. To obtain the neurite attachment points, a Dilate command with the iteration value of 1 and the count value of 1 is used to increase the size of somata. Dilated soma image was combined with skeleton image using the logical operation "AND". The result image, "stem", consists of single-pixel wide objects located within the soma. An Erode command with the iteration value of 1 and the count value of 7 counts followed by a Subtraction command was then used to isolate the tip pixels of these single-pixel wide objects. The attachment points were "stem-point" pixels that do not intersect with somata.

Create an image *I-attachment_points *for neurite attachment point detection

Step 1) *I-soma_dilate *= run("Dilate") on *I-soma*

Step 2) *I-stem *= imageCalculator("And create","*I-soma_dilate*","*I-neuritesoma*")

Step 3) *I-stem_erode *= run("Erode") on *I-stem*

Step 4) *I-stem_points *= imageCalculator("Subtract create"," *I-stem*"," *I-stem_erode*")

Step 5 *I-attachmentpoints *= imageCalculator("Subtract create"," *I-stem_points*","*I-soma*")

Step 6) Quantify attachment points using "Analyze Particles".

### Ending point extraction

We define the ending point as the location at the tip of neurites. An Erode command with the iteration value of 1 and the count value of 7 was used to remove just one pixel from the tip of a filament. To obtain the neurite ending points, the end pixels of the single-pixel objects in the skeleton image were retained and the resulting pixels that do not intersect with dilated soma were assigned as ending points.

Create image *I-endpoints *for ending point detection.

Step 1) *I-neurite_erode *= run("Erode) on *I-neurite_length*

Step 2) *I-tip *= imageCalculator("Subtract create"," *I-neurite_length*","*I-neurite_erode*")

Step 3) *I-end_points *= imageCalculator("Subtract create"," *I-tip*","*I-soma_dilate*")

Step 4) Quantify ending points points using "Analyze Particles".

## Results

### Function and speed comparison between NeurphologyJ and NeuriteTracer

Table 2 shows the comparisons between the two automatic ImageJ-based methods NeurphologyJ and NeuriteTracer [14]. NeurphologyJ can quantify more morphological features from a single image. Because NeuriteTracer needs to load the entire image stack into ImageJ before running the analysis, the number of images can be processed in one batch is limited by the amount of RAM memory. NeurphologyJ uses dynamic memory allocation for batch processing that the memory allocated for processing one image is released immediately after the analysis is over. Therefore, all the images in a folder can be analyzed in one batch. This high-throughput version of NeurphologyJ is designed for ImageJ 1.43 and later. For analysis speed comparison, three test images downloaded from the NeuriteTracer website were analyzed in a same computer using the same ImageJ version. The time needed for analyzing one image is 2.1 and 1.7 seconds using NeuriteTracer and NeurphologyJ, respectively.

**Table 2:** Comparisons between the two automatic ImageJ-based methods NeurphologyJ and NeuriteTracer

Measurements (Function, speed, and accuracy)	NeuriteTracer	NeurphologyJ
Neurite length	Yes	Yes

Soma number	Yes	Yes

Soma size	No	Yes

Attachment point	No	Yes

Ending point	No	Yes

Image for analysis	Pair of images	Single image

No. of Images in one batch	Limited by memory	Unlimited

Analysis speed per image	2.1 sec	1.7 sec

Correlation coefficient/p-value* (primary neurons)	0.97/<0.0001	0.97/0.2696

Correlation coefficient/p-value* (P19 neurons)	0.97/0.0025	0.99/0.5678

* Manual tracings using NeuronJ are used as gold standard for calculating correlation coefficient and p-value.

### Accuracy comparison between NeurphologyJ and NeuriteTracer

**Figure 4:**
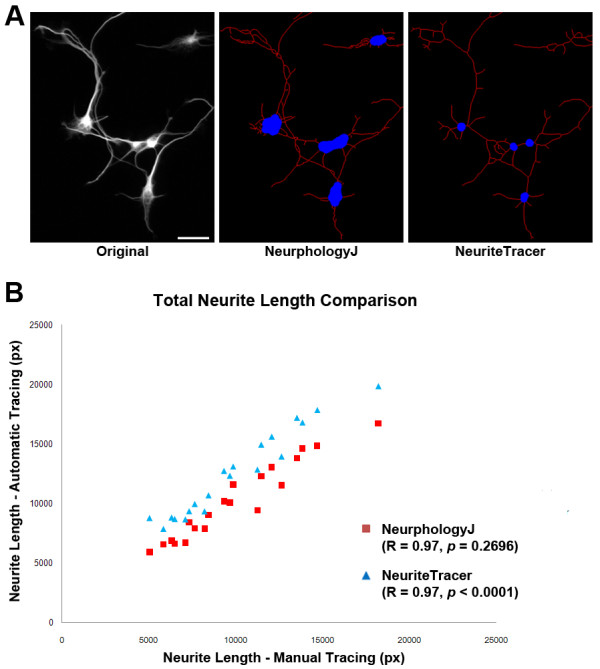
**NeurphologyJ produces accurate neurite length estimation in images of primary hippocampal neurons**. (A) Example image of mouse hippocampal neurons analyzed by NeurphologyJ and NeuriteTracer. Neurite tracings are shown in red and somata in blue in analyzed images. The scale bar represents 50 μm. (B) Twenty hippocampal neuron images manually traced using NeuronJ, or automatically traced using NeurphologyJ and NeuriteTracer were compared. Total neurite length (in pixels) obtained by manual tracing is plotted on the X-axis, and total neurite length obtained by automatic tracing was plotted on the Y-axis. Pearson's correlation coefficients and paired two-tailed Student's t-test are indicated.

To evaluate NeurphologyJ, we compared neurite tracing results with those of NeuronJ and NeuriteTracer. We first analyzed images of mouse hippocampal neurons using these three methods (Figure 4A). These twenty hippocampal neuron images are provided as supplemental image set 1 and can be downloaded from NeurphologyJ website. Manual tracings using NeuronJ are used as gold standard for comparison. Both NeurphologyJ and NeuriteTracer produced tracings that were highly correlated with manual tracings (Pearson's correlation coefficients R = 0.97 for NeurphologyJ and R = 0.97 for NeuriteTracer) (Figure 4B). However, while NeurphologyJ generated neurite tracings are not statistically indistinguishable from manual tracings (*p *= 0.2696 from the paired two-tailed Student's *t*-test), those from NeuriteTracer are statistically different (*p *< 0.0001).

**Figure 5:**
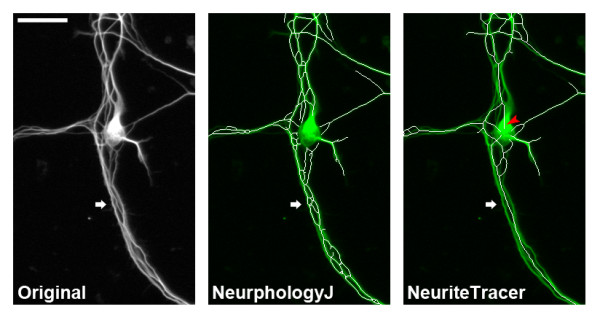
**NeurphologyJ can identify neurites that lie in close proximity to each other**. Mouse hippocampal neurons immunofluorescence stained were analyzed using NeurphologyJ and NeuriteTracer. Neurite tracings are shown in white and the original image is shown in green in merged images. Note that NeuriteTracer falsely identifies the area outside the nucleus as neurites (red arrowhead) and is unable to detect neurites in bundles (white arrows). The scale bar represents 50 μm.

The reason for this difference is the overestimation of neurite length from NeuriteTracer. NeuriteTracer uses DAPI staining to identify somata. This method leads to false identification of the area outside of DAPI-filled nucleus as neurites (Figure 5, red arrowhead). NeurphologyJ takes away the need of the DAPI image and essentially eliminates this problem. Furthermore, NeuriteTracer is unable to distinguish neurites located in close proximity or in bundles (Figure 5, white arrow). The edge detection operation in the enhancement process allows NeurphologyJ to circumvent this problem.

**Figure 6:**
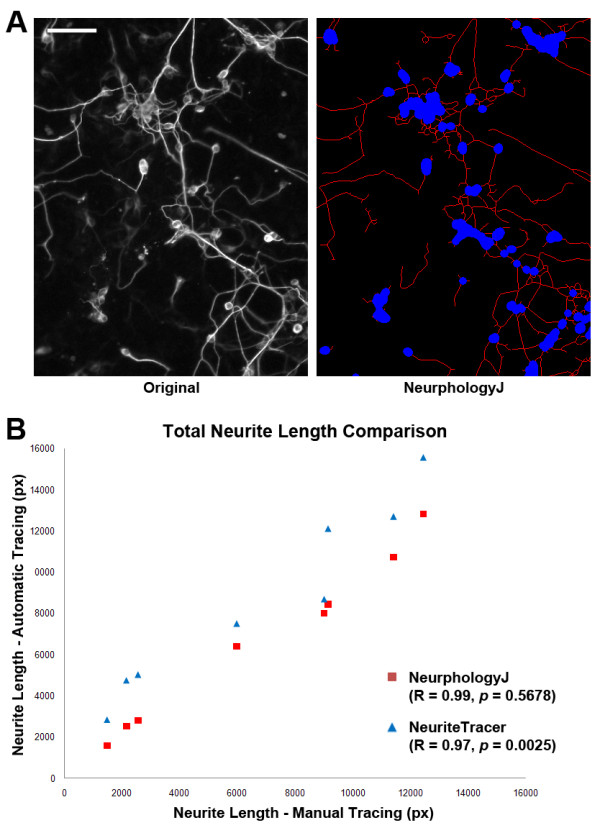
**NeurphologyJ produces accurate neurite length estimation in images of P19 neurons**. (A) Example image of P19 neurons analyzed by NeurphologyJ. Neurite tracings are shown in red and somata in blue in the analyzed image. The scale bar represents 50 μm. (B) Eight images of P19 neurons manually traced using NeuronJ, or automatically traced using NeurphologyJ and NeuriteTracer were compared. Total neurite length (in pixels) obtained by manual tracing is plotted on the X-axis, and total neurite length obtained by automatic tracing was plotted on the Y-axis. Pearson's correlation coefficients and paired two-tailed Student's t-test are indicated.

To test whether NeurphologyJ also performs well in other type of neurons, we analyzed images of neurons differentiated from the embryonic carcinoma P19 cells [27] (Figure 6A). These eight P19 neuron images are provided as supplemental image set 2. Both NeurphologyJ and NeuriteTracer generated neurite tracings that showed excellent correlation with manual tracings (Pearson's correlation coefficients R = 0.99 for NeurphologyJ and R = 0.97 for NeuriteTracer) (Figure 6B). NeurphologyJ generated neurite tracings are not statistically different from manual tracings (*p *= 0.5678), while those from NeuriteTracer are statistically different (*p *= 0.0025).

**Figure 7:**
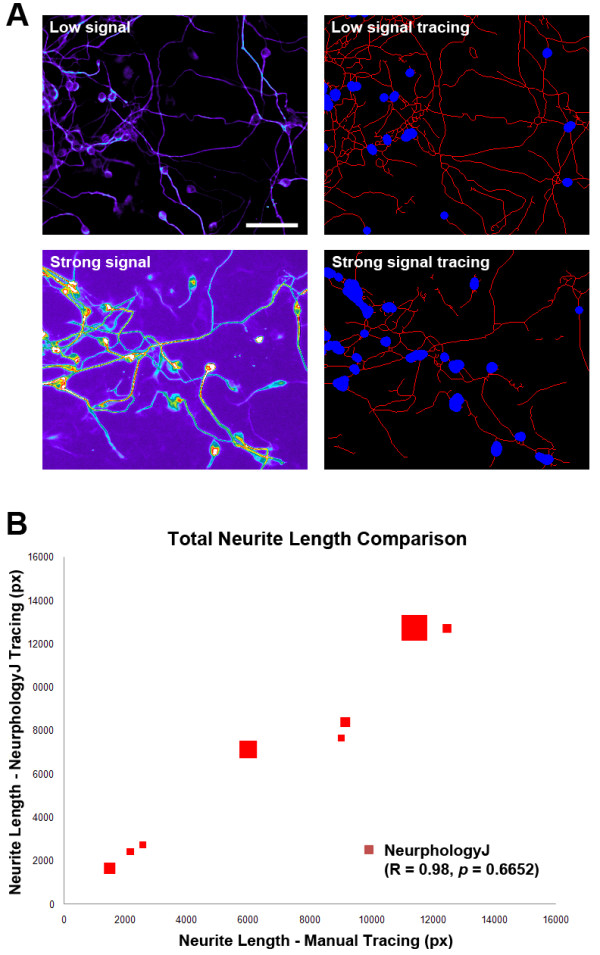
**NeurphologyJ produces reliable neurite quantification in images with different signal intensities**. (A) Examples of images with different signal intensity analyzed by NeurphologyJ. Original images are rendered in pseudo-color to visualize signal intensity. Neurite tracings are shown in red and somata in blue in analyzed images. The scale bar represents 50 μm. (B) Eight P19 neuron images with varying signal intensity were manually traced using NeuronJ or automatically traced using NeurphologyJ. The resulting total neurite lengths were compared. Each data point consists of three parameters: the X-axis value represents the neurite length (in pixels) obtained by manual tracing; the Y-axis value represents the neurite length obtained by NeurphologyJ, and the size of each data point is in proportion to the average signal intensity of that image. Pearson's correlation coefficients and paired two-tailed Student's t-test are indicated.

To test whether NeurphologyJ is tolerable to signal variation, we analyzed eight images of P19 neurons with varying signal intensity. Eight P19 neuron images were manually traced using NeuronJ, or automatically traced using NeurphologyJ, and the resulting total neurite lengths were compared. In Figure 7, each data point consists of three parameters: the X-axis value represents the neurite length (in pixels) obtained by manual tracing; the Y-axis value represents the neurite length obtained by NeurphologyJ, and the size of each data point is in proportion to the average signal intensity of the corresponding image. Despite varying signal intensity, NeurphologyJ generates neurite tracings that are highly correlated (R = 0.98, Pearson's correlation coefficient) and statistically indistinguishable (*p *= 0.6652, paired two-tailed Student's t-test) from manual tracings. These results show that NeurphologyJ can produce accurate neurite tracings that are comparable to those obtained manually. In addition, NeurphologyJ is quite forgiving towards signal variation and thus making it a reliable analytical tool for high-content screenings.

### Identifying somata using only neurite staining

**Figure 8:**
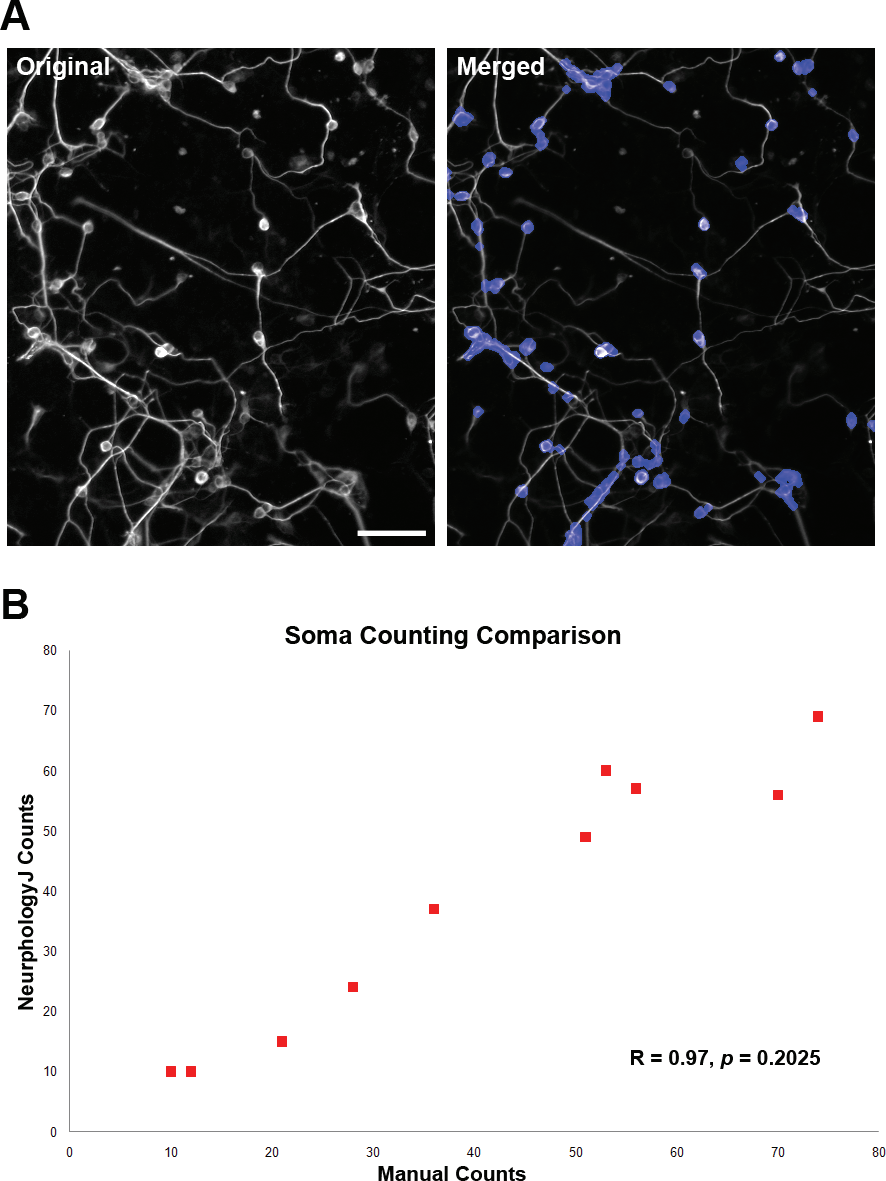
**NeurphologyJ produces accurate soma counts**. (A) An example of soma quantification on P19 neurons using NeurphologyJ. The merged image shows the NeurphologyJ identified somata in blue and the original single-channel image in gray. The scale bar represents 50 μm. (B) A comparison of soma number using manual counting and NeurphologyJ automatic counting. Ten images of TUJ1 stained P19 neurons were used for this comparison. Soma counts obtained manually were plotted on the X-axis, and soma counts obtained by NeurphologyJ were plotted on the Y-axis. Pearson's correlation coefficients and paired two-tailed Student's t-test are indicated.

To test the applicability of NeurphologyJ on soma detection, ten images of P19 neurons stained with neuron-specific antibody (TUJ1) were analyzed (Figure 8A). Somata identified by NeurphologyJ were compared to those manually identified (Figure 8B). Each data point consists of two parameters: the X-axis value represents the soma count obtained manually, and the Y-axis value represents the soma count obtained by NeurphologyJ. NeurphologyJ produced soma counts that were highly correlated (R = 0.97, Pearson's correlation coefficient) and statistically indistinguishable (*p *= 0.2025, paired two-tailed Student's t-test) from those obtained manually.

When multiple somata are located in close proximity, they tend to be counted as one. This produces an underestimation of soma count in images with overcrowded somata. For this reason, NeurphologyJ also generates quantitative data on total soma area. This data allow users to obtain more accurate results of soma quantification. NeuriteTracer is unable to detect somata in P19 neurons because these neurons are required to grow on top of a monolayer of non-neuronal cells. When DAPI is used, nuclei of every cell (both neurons and non-neuronal cells) are stained.

### Quantifying attachment points and ending points

**Figure 9:**
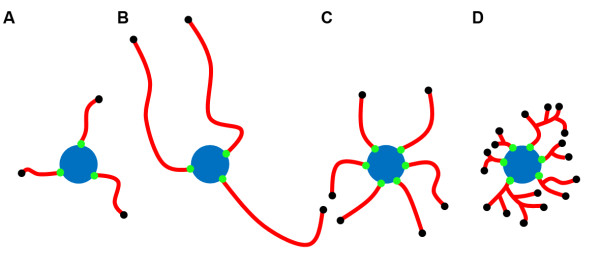
**Neurite complexity can be deduced from neurite attachment point and ending point**. Examples of neurons with different levels of neurite complexity are shown. Note that neuron A and neuron B can be distinguished by total neurite length (NL). Neuron B and neuron C can be distinguished by attachment points (AP). Neuron C and neuron D can be distinguished by ending points (EP). In these examples, NL_A _< NL_B _= NL_C _= NL_D_, AP_B _< AP_C _= AP_D_, and EP_C _< EP_D_.

**Figure 10:**
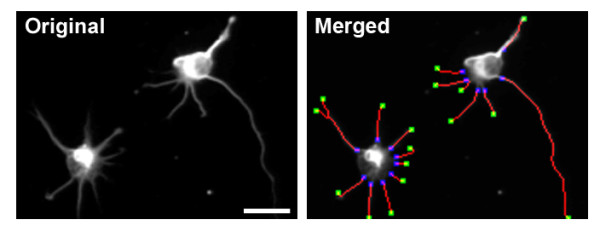
**An example of hippocampal neuron image analyzed using NeurphologyJ**. NeurphologyJ produces accurate neurite analysis of attachment point and ending point (also refer to Tables 3 and 4). Neurites are shown in red, ending points in green, and attachment points in blue in the merged image. The scale bar represents 20 μm.

We defined neurite attachment points as the location where neurites connect to the soma and neurite ending points as the location at the tips of neurites. These two morphological parameters are important because the number of attachment points indicates the number of neurites for a given neuron, and the ratio of attachment point number to ending point number specifies the extent of neurite branching. The higher the attachment points, the more numerous a neuron sprouts neurites. The higher the ratio, the more branches a neuron contains (Figure 9). To determine if NeurphologyJ can provide accurate quantification of neurite attachment points and ending points, we analyzed seven images of mouse hippocampal neurons using NeurphologyJ. Figure 10 illustrates an example image analyzed with NeurphologyJ. These seven images are provided as supplemental image set 3.

For attachment point detection, an average accuracy (dividing correctly assigned attachment points detected using NeurphologyJ by total attachment points manually assigned) exceeds 98%; and an average error rate (dividing incorrectly assigned attachment points detected using NeurphologyJ by all attachment points detected using NeurphologyJ) of 9% can be achieved (Table 3). For ending point detection, the average accuracy exceeds 88% and the average error rate falls around 35% (Table 4). While NeurphologyJ produces fairly good estimation of attachment and ending points, it is more accurate at detecting attachment point than at detecting ending points (compare Table 3 with Table 4).

**Table 3:** Quantification of neurite attachment points using NeurphologyJ

Image id	Manual (Ma)	NeurphologyJ (Ne)	TP	FP	FN	Error Rate*(%)	Accuracy*(%)
1	15	15	15	0	0	0	100

2	12	16	12	4	0	25	100

3	12	14	11	3	1	29	92

4	9	10	9	1	0	10	100

5	13	13	13	0	0	0	100

6	11	11	11	0	0	0	100

7	12	12	12	0	0	0	100

Average						9.08	98.81

*Error rate = (FP+FN)/Ne; Accuracy = TP/Ma

**Table 4:** Quantification of neurite ending points using NeurphologyJ

Image id	Manual (Ma)	NeurphologyJ (Ne)	TP	FP	FN	Error Rate* (%)	Accuracy* (%)
1	17	17	17	0	0	0	100

2	14	19	10	9	4	68	71

3	15	29	14	15	1	55	93

4	9	12	9	3	0	25	100

5	14	14	12	2	2	29	86

6	12	12	11	1	1	17	92

7	17	19	13	6	4	53	76

Average						35.21	88.37

*Error rate = (FP+FN)/Ne; Accuracy = TP/Ma

### Quantifying the effect of nocodazole on P19 neurons

**Figure 11:**
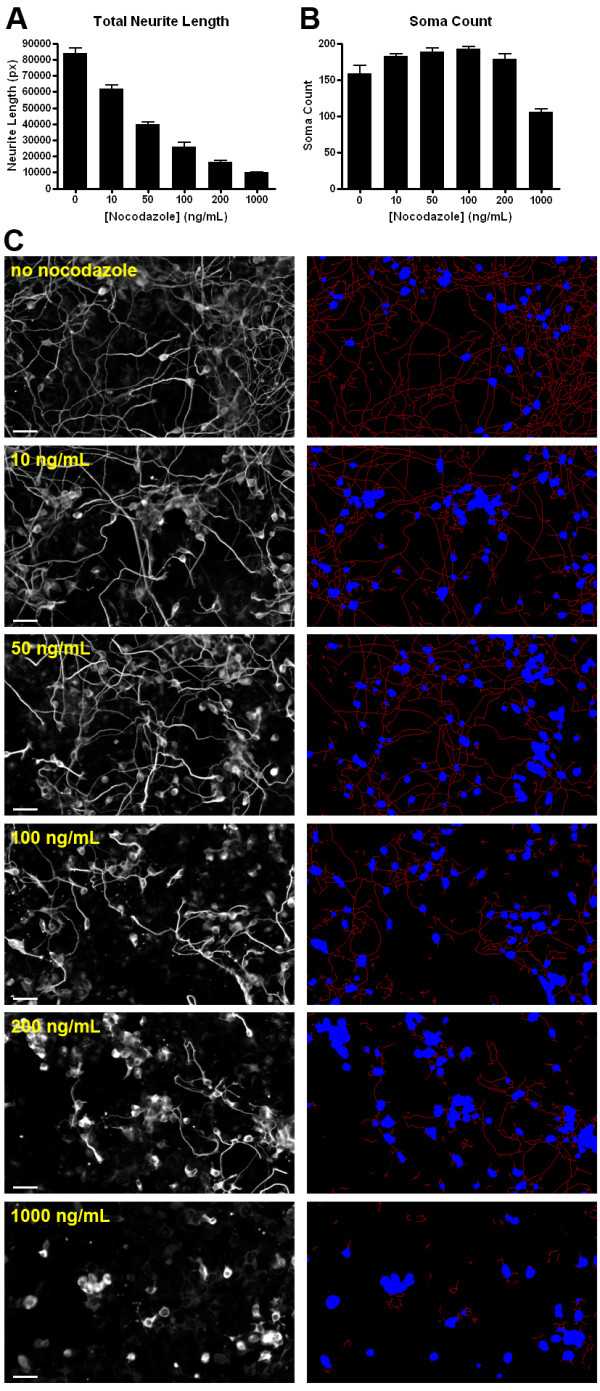
**NeurphologyJ can quantify the neurite length reduction effect of nocodazole**. (A) Bar graph showing the inverse relationship between the total neurite length and nocodazole concentration in P19 neurons. (B) Bar graph showing the concentration of nocodazole does not affect the soma count in P19 neurons unless a high dosage of nocodazole is added. Each bar consists of three independent experiments, and each experiment is derived from 12 different images. Error bars represent standard error of the mean. (C) Typical images of P19 neurons at various nocodazole concentrations. Original images are shown on the left and the analyzed images are shown on the right. Neurite tracings are shown in red and somata are shown in blue. The scale bars in the original images represent 50 μm.

To determine if NeurphologyJ can detect neuronal morphological changes upon pharmacological perturbation, we designed an experiment to measure the effect of nocodazole on neurite length. Nocodazole is a known microtubule-destabilizing drug that has been shown to induce rapid neurite retraction when applied to neurons [25,26]. P19 neurons were incubated with increasing dosage of nocodazole for 24 hrs before being fixed and immunofluorescence stained. A total of 216 images (with a total size over 500 Mb) were analyzed using NeurphologyJ, and the entire analysis was completed around 7 min. For comparison, each image takes over 30 min to analyze by hand. When NeurphologyJ analysis was completed, an inverse correlation can be observed between the neurite length and the dosage of nocodazole (Figure 11A and 11C). This result is in agreement with the function of nocodazole on inducing neurite retraction. Furthermore, the nocodazole dosage-dependent neurite length reduction demonstrates the high sensitivity of NeurphologyJ analysis.

When fitted to a sigmoidal dose-response curve, we were able to calculate the IC50 of nocodazole on neurite outgrowth to be 42 ng/mL. While nocodazole have been extensively used for inducing neurite retraction [26,28-31], the effective concentration has never been systematically determined. To our knowledge, this is the first time the IC50 of nocodazole on neurite outgrowth is determined. The soma count, on the other hand, was not significantly altered until the highest dosage of nocodazole (1000 ng/mL) was applied (Figure 11B). This result agrees nicely with the observation that nocodazole at high dosage activates the JNK/SAPK signalling pathway and induces apoptosis [32,33]. Taken together, these results demonstrate the applicability of NeurphologyJ in detecting neuronal morphological changes upon drug treatment.

## Discussion

We have tested and validated the applicability of NeurphologyJ using two types of neurons: primary neurons from the dissociated hippocampal culture and the cell line derived P19 neurons. It is very likely that NeurphologyJ can be applied to other types of neurons. Potentially, NeurphologyJ can be applied to quantify other biological structures that are fibrous in shape, e.g. blood vasculature and fungal hyphae. We benchmarked NeurphologyJ and found that a typical 1360 × 1032 pixel dimension image takes roughly 1.7 seconds to complete. This is a significant improvement over manual tracing and is slightly better than the performance of another automatic tracing method NeuriteTracer.

It is important to note that NeurphologyJ operates on the entire image. When an overlapping neurite network has established (see Figure 11C for example), it is extremely difficult if not impossible to identify the origin of a particular neurite. This is the reason why NeurphologyJ was not developed to quantify neuronal morphology on a per cell basis. If the average neurite length for neurons is needed, users can easily obtain it by dividing the total neurite length with total soma count or total soma area.

**Figure 12:**
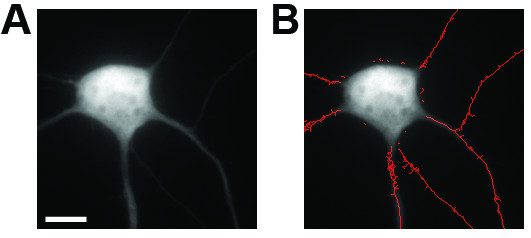
**NeurphologyJ produces tree-like tracings when high magnification images are used**. Shown here is (A) an example image of a mouse hippocampal neuron acquired using a 60x 1.35 N.A. objective lens and (B) the NeurphologyJ-traced image. Notice the tree-like tracings in the neurites. The scale bar represents 20 μm.

One limitation of NeurphologyJ is that it does not quantify neurite length accurately on images acquired using high magnification objectives (equal or higher than 40x). This is because the neurite width in high magnification images is usually quite large. When Skeletonize operation is applied to these wide neurites, it produces tree-like skeletons, and this result in an overestimation of the neurite length (Figure 12).

We should also point out that the accuracy of neurite ending point detection depends on the nature of the image. NeurphologyJ cannot perform correct ending point quantification on neurons with highly fragmented neurites, such as neurons undergoing apoptosis. This is because the tips each neurite fragment will be falsely recognized as ending points. It is possible to remedy this by using various filling and pruning strategies [34-36].

**Figure 13:**
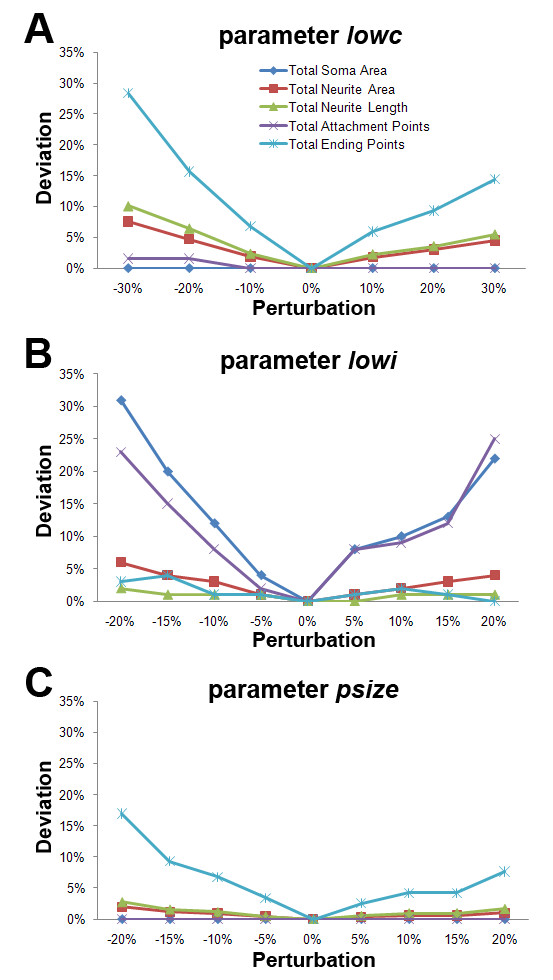
**Robustness of each user-defined parameter on a typical image Robustness**. The user-defined parameters are individually perturbed while keeping the other two constant. (A) Parameter *lowc *(the expert user determined value is 10) (B) Parameter *lowi *(the expert user determined value is 260) (C) Parameter *psize *(the expert user determined value is 20). The X-axis shows the perturbation from the expert user-defined value and the Y-axis shows the deviation of the resulting quantification outputs from the 0% perturbation-derived ones. It is important to note that the flatter the lines, the more robustness the parameter is.

There are four independent and user-defined parameters in NeurphologyJ (*lowc*, *lowi*, *nwidth*, and *psize*). We have included a user manual to help user determine the values of these parameters in a logical manner (Additional File 1). Since the value of the parameter *nwidth *is equal to the width of the thickest neurite, it can be readily determined from users' own images. Therefore, we evaluated the robustness of the other three user-defined parameters by perturbing these expert user determined values. The results of using a typical image are given (Figure 13). Judging from our analyses, the parameter *lowi *most strongly influenced the morphological quantification (Figure 13B), whereas *psize *is the most robust parameter (Figure 13C) and *lowc *is the second robust parameter (Figure 13A). In addition, the total neurite length and total neurite area quantifications appeared to be the most reliable outputs. At 10% perturbation, all except one (total soma area) of our quantification outputs are within 10% of deviation. In summary, NeurphologyJ is a robust quantification method able to forgive moderate amount of perturbation.

## Conclusions

We have proposed an automatic neuronal morphology quantification method and its ImageJ plugin named NeurphologyJ with supports for single image (interactive version) or batch (high-throughput version) processing. The utilization of the freeware tool NeurphologyJ allows rapid, consistent, and objective quantification on soma number, soma area, neurite length, attachment point, and ending point. We applied NeurphologyJ to a high-content screen and successfully determined the IC50 of nocodazole on neurite outgrowth to be 42 ng/mL.

## Availability

The source codes of NeurphologyJ (interactive and high-throughput versions) and the images used for testing are freely available. Mouse hippocampal neuron images used for quantifying neurite length are provided as supplemental image set 1. P19 neuron images used for quantifying neurite length and soma number are provided as supplemental image set 2. Mouse hippocampal neuron images used for quantifying neurite attachment points and ending points are provided as supplemental image set 3. We have also set up a website for accessing all the files mentioned above at http://life.nctu.edu.tw/~microtubule/neurphologyJ.html

## Competing interests

The authors declare that they have no competing interests.

## Authors' contributions

SYH and EH designed the system, participated in manuscript preparation, and carried out the detail study. CYC and EH conducted all experiments. HLH, PC and EH implemented programs. SYH, HLH, TWC, and EH conceived the idea of this work. EH supervised the whole project. All authors have read and approved the final manuscript.

## Supplementary Material

Additional File 1 **User manual**. This PDF file is the user's manual for NeurphologyJ interactive and high-throughput versions. It walks the users through step by step. Click here for file
