# Supplementary material for: NeurphologyJ: An automatic neuronal morphology quantification method and its application in pharmacological discovery
Source: BMC Bioinformatics. 2011 Jun 8;12:230. doi: 10.1186/1471-2105-12-230 (PMC3121649; doi:10.1186/1471-2105-12-230)
Supplement: Additional File 1 — User manual. This PDF file is the user's manual for NeurphologyJ interactive and high-throughput versions. It walks the users through step by step. [file 1471-2105-12-230-S1.PDF]

[Installation Instruction]

1. Download and install ImageJ (a version newer than "1.43u" is required).

2. Unzip and place all files into the "plugins" folder of ImageJ (default - C:\Program Files\ImageJ\plugins)

3. NeurphologyJ interactive version requires an additional ImageJ plugin called "Particle Remover", which can be downloaded from the following website:
   http://rsb.info.nih.gov/ij/plugins/particle-remover.html

4. Start ImageJ and you should see NeurphologyJ option under the Plugins menu.

[Operation Instruction: NeurphologyJ interactive]

1. Click on NeurphologyJ interactive and the plugin will ask you to select an image. Click OK and a window will show up and ask you to locate the image.

   NeurphologyJ will take images up to 16-bit. We recommend using images 12-bit or higher. In addition, NeurphologyJ only works on fluorescent or darkfield images that have dark background and high signal-to-background ratio.

   We recommend analyzing neuronal morphology using images acquired with a 10x objective lens. This produces the most reliable neurite length measurement. Images acquired using 40x lens or higher may cause image processing problem.

2. After the image is loaded, NeurphologyJ prompts you whether a set-up wizard is needed. This wizard will help you optimize the image and is strongly recommended for first-time user. Click Yes to proceed.

3. In the first threshold selection window, click the "Dark background" option.

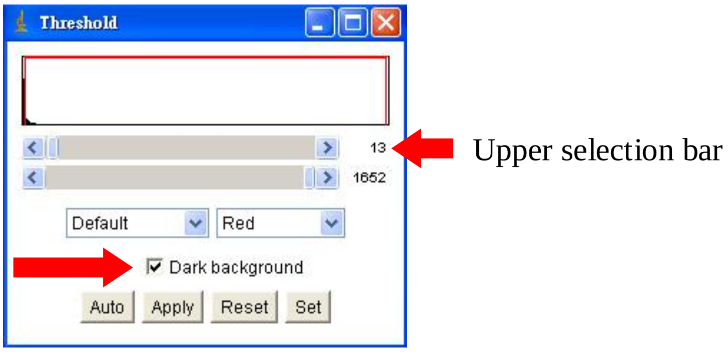

4. Move the upper selection bar so that all of the neurites (or cellular protrusions) are selected.

An image with optimal threshold is shown below. Don’t worry if some background pixels are selected, they will be removed at a later step.

Optimal threshold:

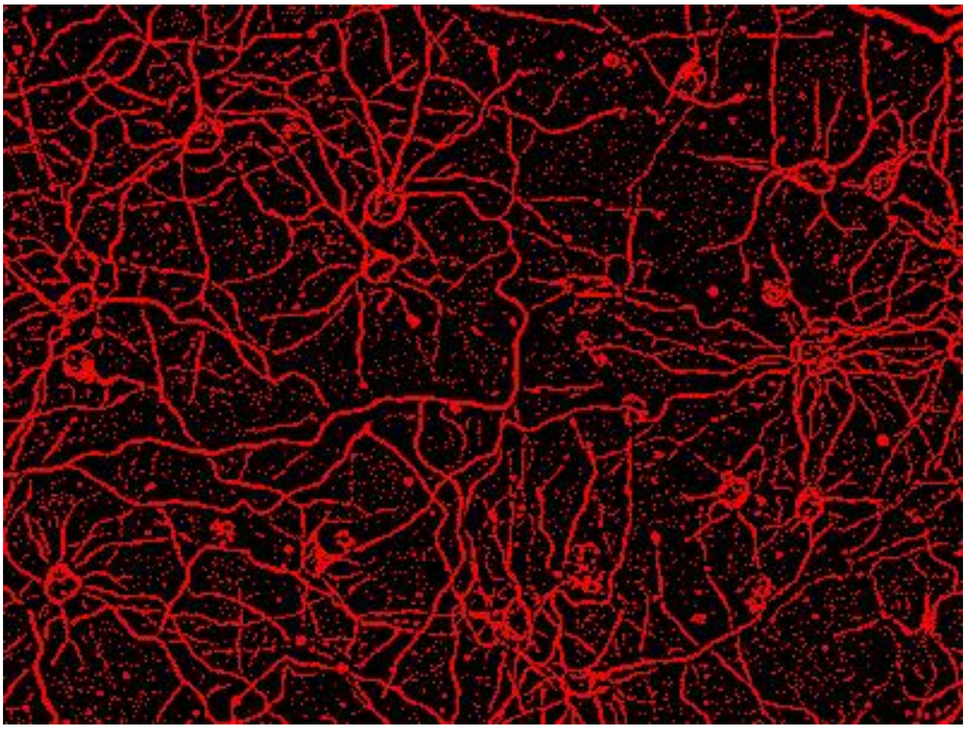

The image below has the threshold value set too high:

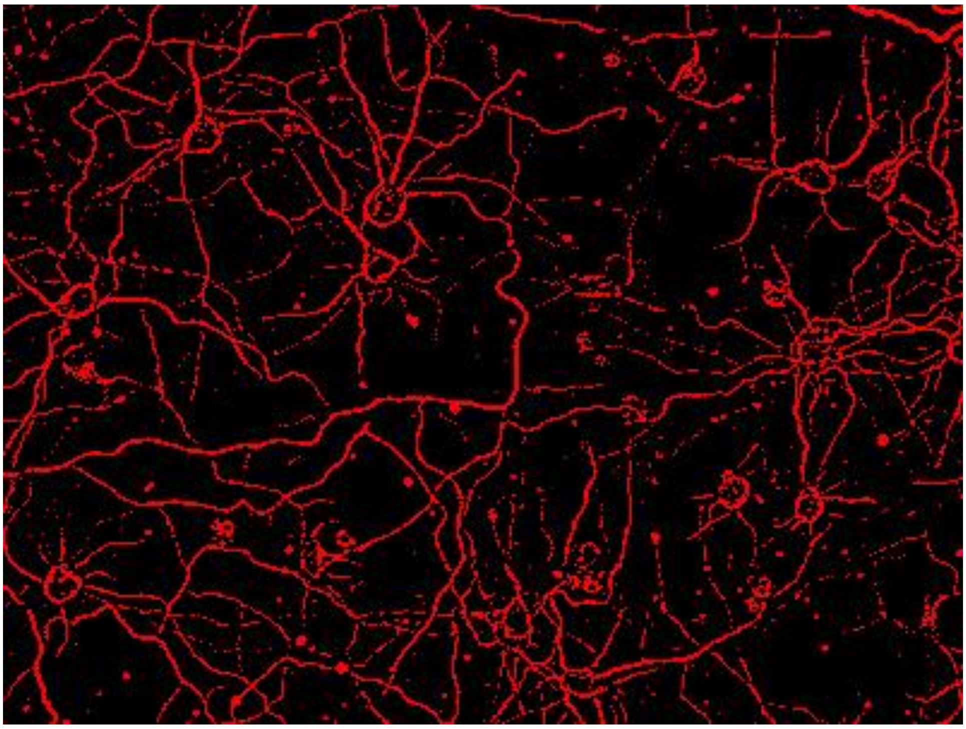

The image below has the threshold value set too low:

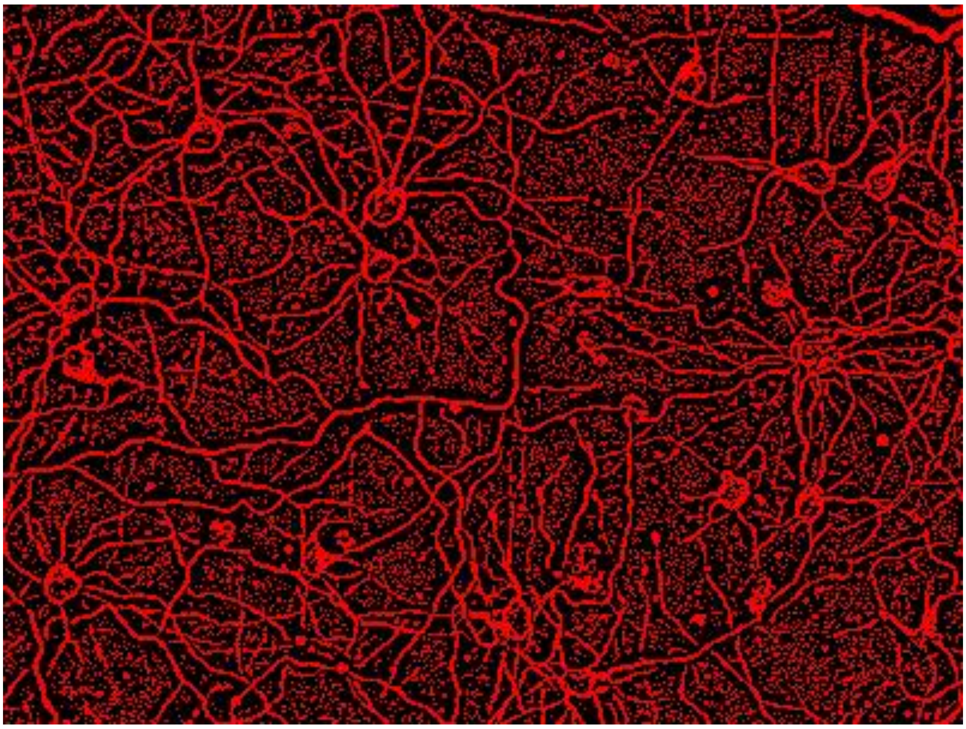

5. Click OK when optimal threshold is achieved. This selection window might be hidden behind other windows, so look for it.

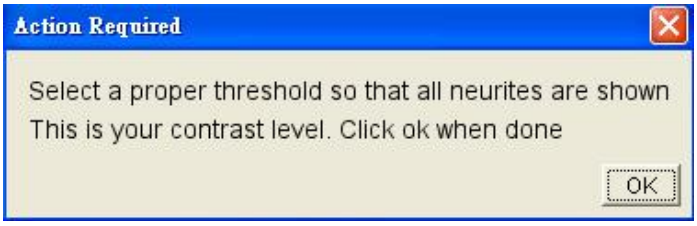

6. Next, you will need to select an optimal threshold for the cell body. Move the upper selection bar so that all cell bodies are selected. It is okay that some of the neurites become selected. An image with optimal threshold is shown below.
   Optimal threshold:

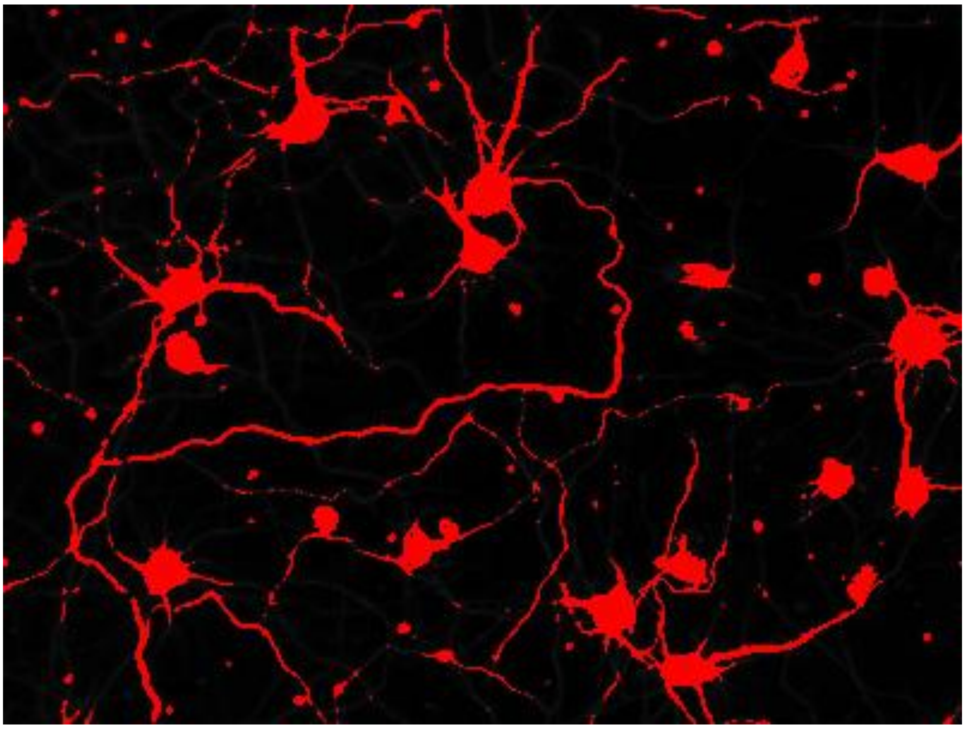

The image below has the threshold set too high:

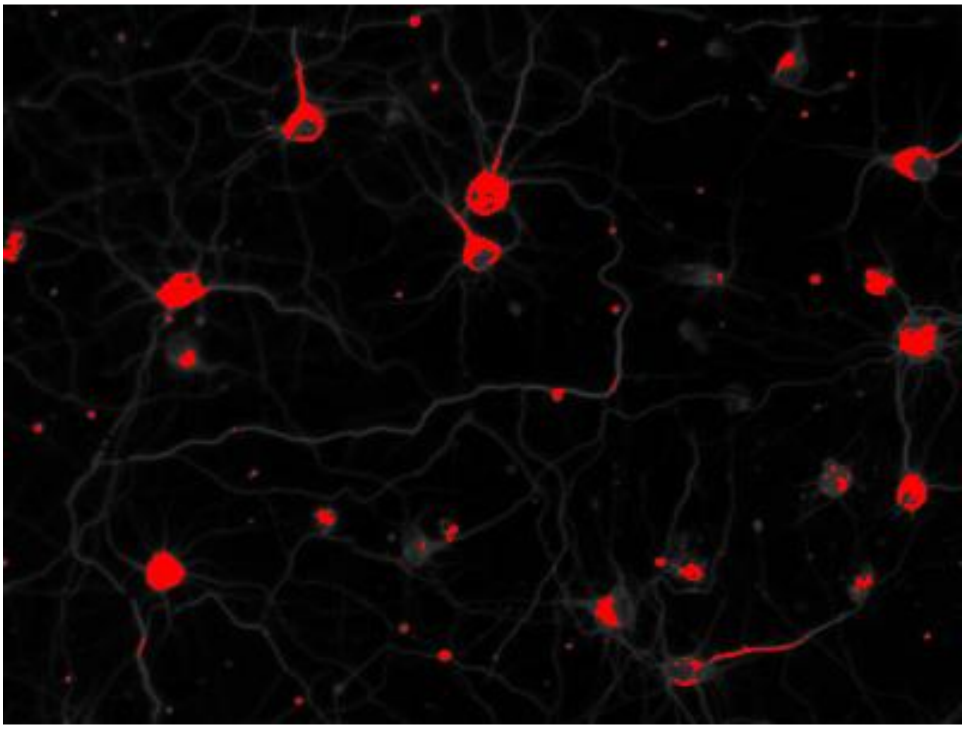

The image below has the threshold set too low:

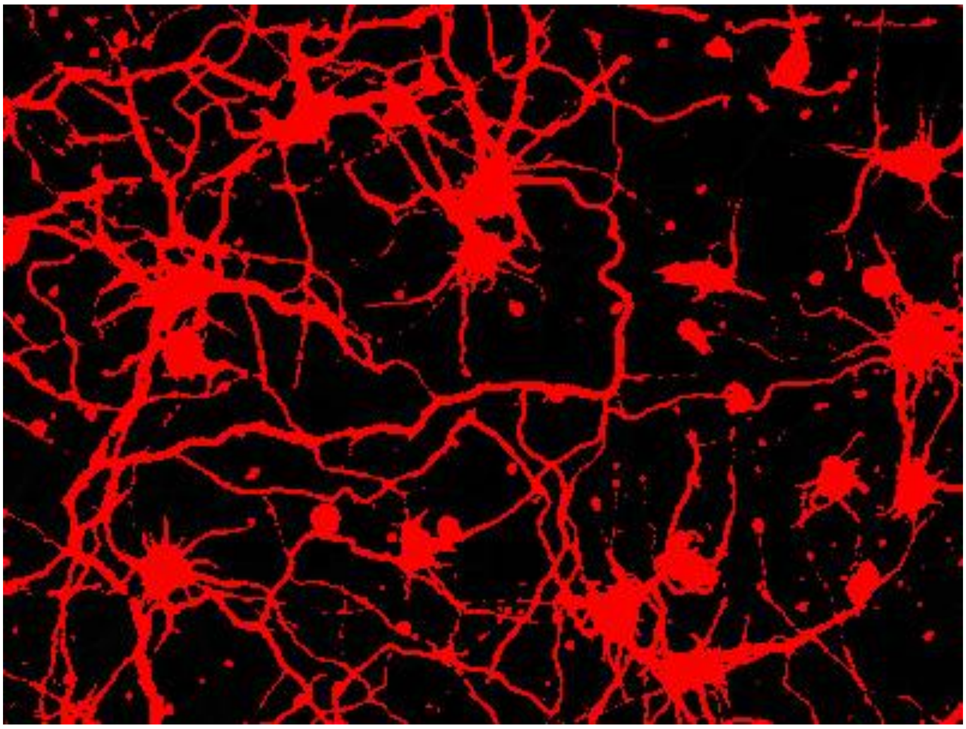

7. After this set-up wizard is done, the window will show up asking for the “contrast level”. Enter the contrast level value as shown in the log window. The log window might be hidden behind other windows, so look for it. Click OK when done.

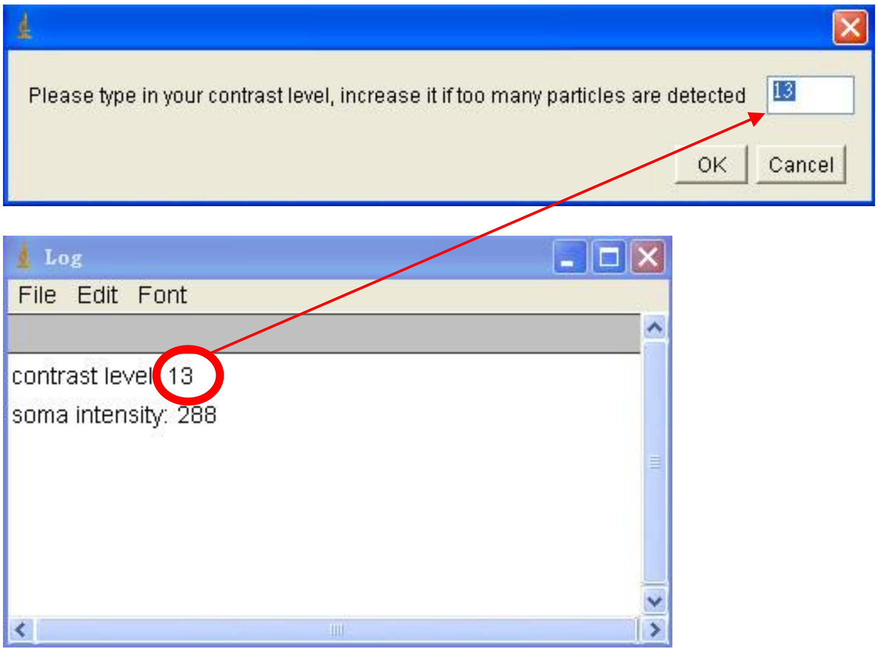

8. Next the algorithm will ask for the soma intensity. Enter the soma intensity value as shown in the log window. Click OK when done.

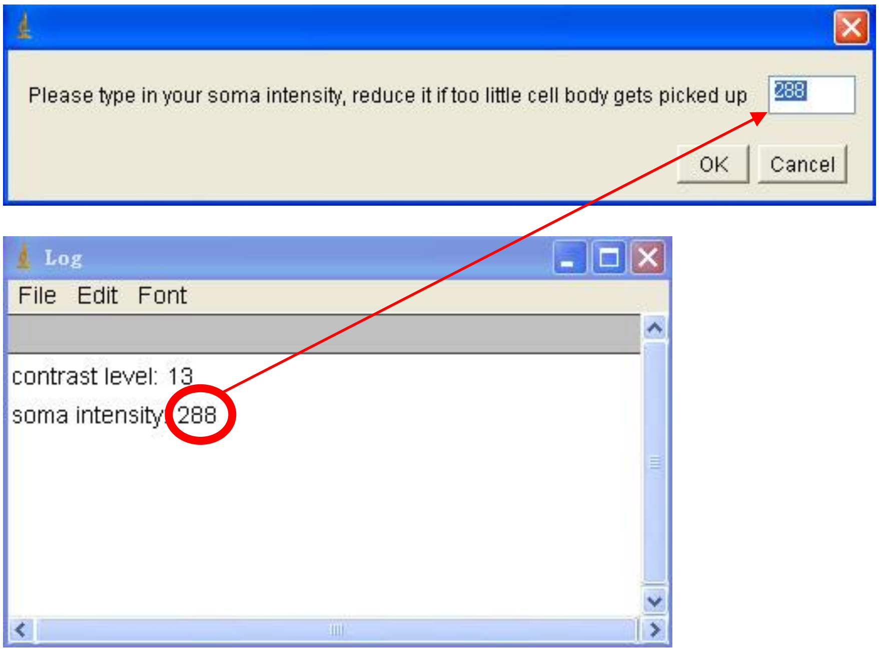

9. The next window ask for the neurite (or cell protrusion) width in pixel. Enter the correct value and click OK to proceed.

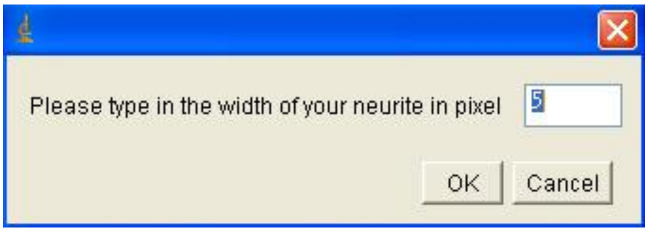

10. The final user-defined window shows up and asks for "particle cleanup value". This value (in pixel) determines the maximal size of particles to be removed from your images. If your images contain tiny little cell debris or dye aggregates, try value of 15 to start with. Increase this value to remove larger objects. After clicking OK, NeurphologyJ will proceed through a number of image operations.

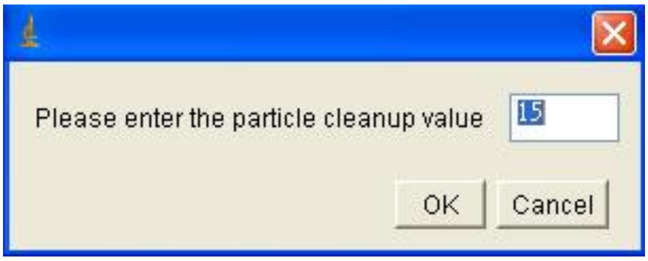

11. Afterwards, a window will show up and ask for a proper threshold. Move the upper selection bar so that it falls within the region without pixel counts. The exact position of the selection bar does not matter as long as it is in the region without pixel counts. Click OK when done.

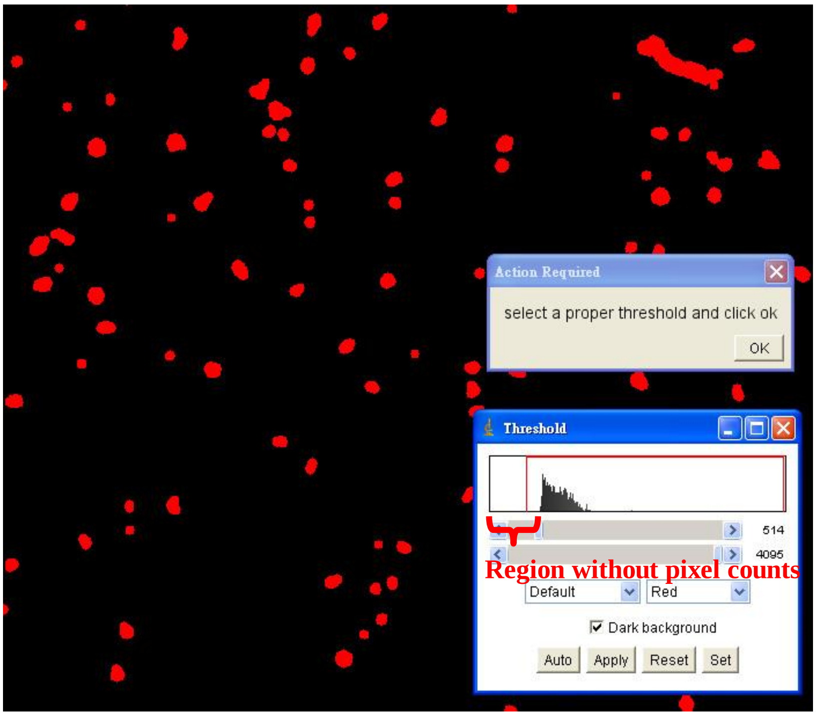

12. Finally, a window will show up and ask for another threshold. Again, move the upper selection bar so that it falls within the region without pixel counts. Click OK when done.

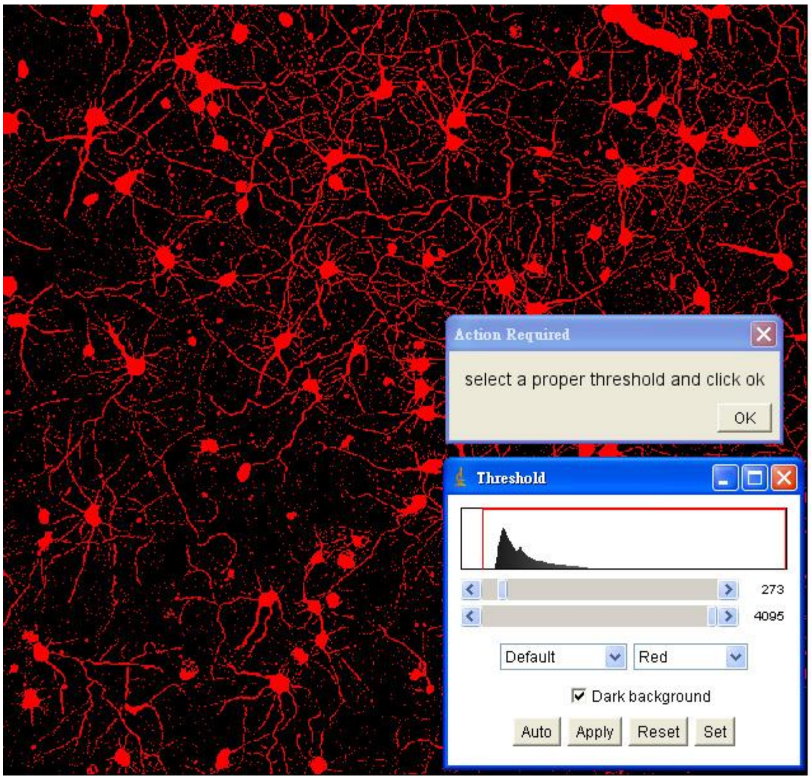

13. NeurphologyJ runs through the last phase of the operation and generates 3 image windows along with a summary table. The header of the image windows indicates the analyzed morphological parameters. Values under the "Total Area" column show different morphological parameters in the unit of pixel. Use the values under the "Count" column with caution (you can potentially underestimate a certain morphological feature).

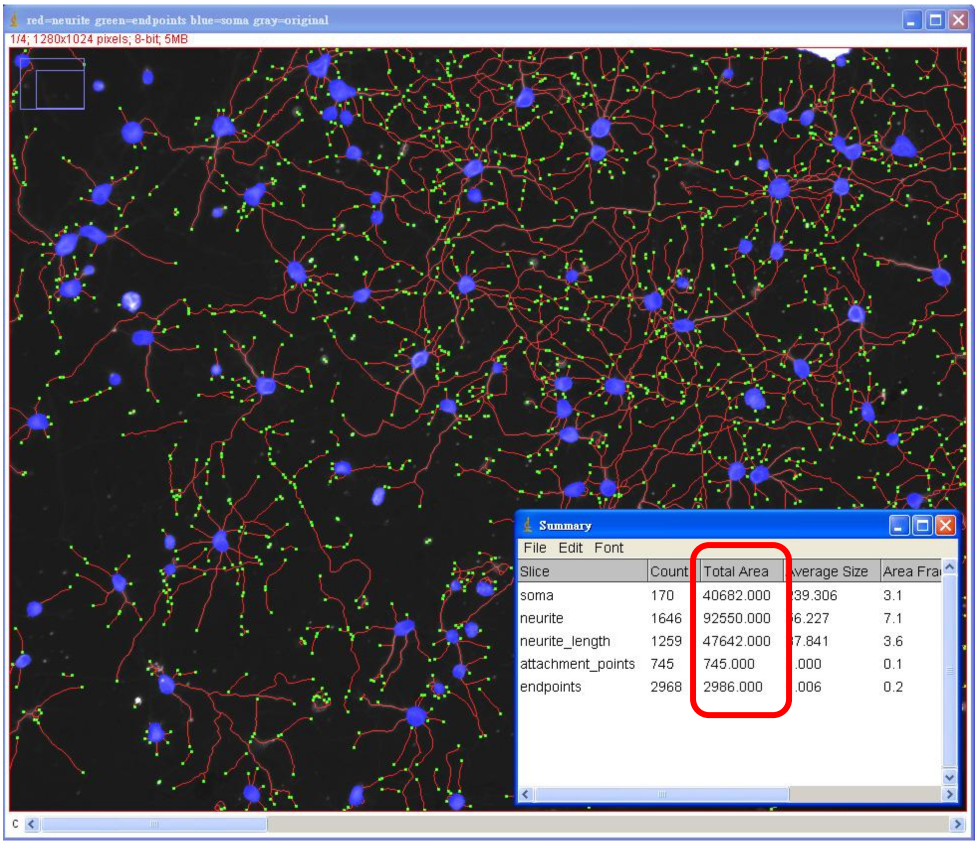

You can use the "Channels" tool to activate (or deactivate) certain image channels.

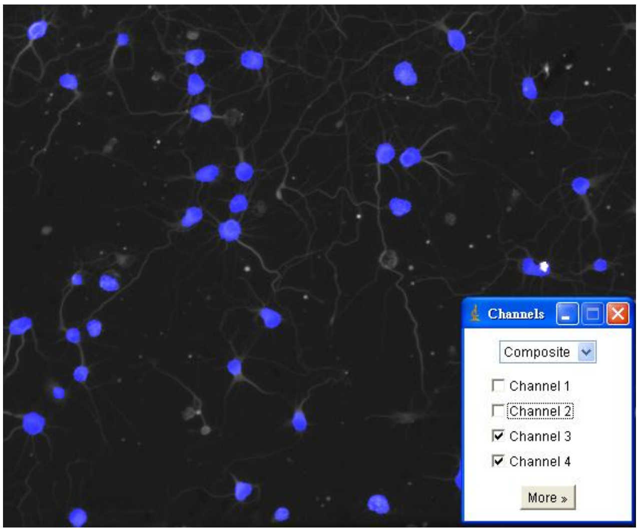

14. (**Note**) NeurphologyJ quantifies the morphological parameters on the entire image field basis. In order to derive the average neurite length per neuron, you will need to divide the “neurite_length” parameter by “soma” parameter.

15. (**Note**) The “neurite” parameter measures the total area covered by the neurites, whereas the “neurite_length” parameter measures the length of the neurites. As a result, you can derive the neurite width via dividing “neurite” parameter” by “neurite_length” parameter.

[Operation Instruction: NeurphologyJHT]

1. Click on NeurphologyJHT and the algorithm will ask you to choose an image directory. Select the folder which contains all your images and click OK.

2. The next window will ask you to enter 4 parameters:

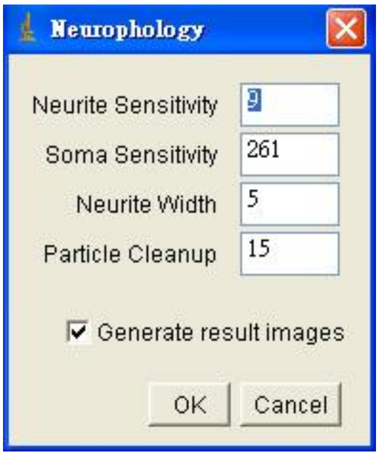

3. Enter the “contrast level” value determined from the interactive version into “Neurite Sensitivity”.
   Enter the “soma intensity” value determined from the interactive version into “Soma Sensitivity”.
   Enter the width of a typical neurite in “Neurite Width”.
   Enter the “particle cleanup value” determined from the interactive version into “Particle Cleanup”.

4. If “Generate result images” is clicked, NeurphologyJHT will also generate images consist of neurite tracings (red), neurite attachment points or ending points (green), and somata (blue).

5. After clicking OK, NeurphologyJHT will analyze all images in the folder you specified.

6. When the analysis is done, the time spent will be displayed.

7. The “Result.txt” and all analyzed images will be stored in the same folder as the original images.
